# Self-Assessed Driving Skills and Risky Driver Behaviour Among Young Drivers: A Cross-Sectional Study

**DOI:** 10.3389/fpsyg.2022.840269

**Published:** 2022-04-13

**Authors:** Timo Lajunen, Mark J. M. Sullman, Esma Gaygısız

**Affiliations:** ^1^Department of Psychology, Norwegian University of Science and Technology (NTNU), Trondheim, Norway; ^2^Department of Social Sciences, University of Nicosia, Nicosia, Cyprus; ^3^Department of Economics, Middle East Technical University, Ankara, Turkey

**Keywords:** risk, young drivers, learning, safety skills, perceptual-motor skills, driver behaviour

## Abstract

The first few years of driving is a critical period when driving skills develop and the driving style is established. While the actual driving skills improve during the first few years of driving, a novice driver’s view of himself/herself as a safe and/or skilful driver also develops rapidly. The aim of this study was to investigate self-evaluated driver safety and perceptual-motor skills among different age groups of young drivers, along with the relationships between self-evaluated skills and driving behaviour. The sample consisted of a stratified random sample of 18–25-year-old drivers from the Finnish driving licence register. The questionnaires, which included the Driver Skill Inventory (DSI), Driver Behaviour Questionnaire (DBQ) and background information, were completed and returned by a total of 1,058 participants. While female drivers assessed their safety skills to be higher than their perceptual-motor skills, the opposite was true for males. In both sexes, perceptual-motor skills increased, and safety skills decreased with experience. Perceptual-motor skills correlated negatively with safety skills, lapses and errors, but positively with aggressive and ordinary violations. Safety skills correlated negatively with all DBQ variables. Safety orientation seems to be most clearly reflected in deliberate aberrant driving behaviours. Sex differences were observed in the development of behaviours and skills, perceptual-motor skills only increased with age among males, while safety skills decreased through experience among both men and women. Results showed that driving experience was strongly related to both driving style (violations, errors) and the drivers’ view of their skills (safety orientation), highlighting the importance of the first few years of driving.

## Introduction

Young road users (aged 18–24 years old) are more likely to die in traffic accidents than any other age group worldwide (European Commission, 2018). In the European Union, the percentage of fatalities among young road users was 12% in 2019, even though this group only makes up 8% of the population (European Commission, 2021). The overrepresentation of young—primarily male—drivers does not seem to depend on a countries general level of traffic safety. For example, the proportion of young people is even higher in European countries like Sweden (16.3%), the United Kingdom (15.0%) and Finland (16.0%), which rank among the safest countries in the world, in terms of traffic safety (European Commission, 2018). In Finland, the road fatality rate, in proportion to the population, was 4.7 fatalities per 100,000 vehicles in 2016. The same figures for 18–20 year olds and 21–24 year olds were 11.1 and 7.3, respectively (Suomen virallinen tilasto, 2019). In the same year, of the 5,911 injured in Finnish traffic, 584 road users were 18–20 year olds, and 481 road users were 21–24 year olds (Suomen virallinen tilasto, 2019). These figures show that young road user injuries and fatalities continue to be an unresolved problem, even in the safest countries, and despite the positive developments in general road safety.

The higher risk of young drivers is also reflected in behaviour. The analysis of accident causes shows that young drivers differ from older (>24 years) drivers in speeding, driving faster than appropriate for the situation, and travelling at speed unexpected by other road users (European Commission, 2018). Driving in the wrong direction was also recorded more frequently among younger drivers (European Commission, 2018). ‘Incorrect direction’ refers to situations in which a manoeuvre was carried out in the wrong direction or involved driving off the road. The analyses of young driver accidents in Finland also showed that exceeding the speed limit by more than 10 km/h, driving under the influence of alcohol, losing control of the vehicle, and not using seat belts were more common among young people involved in a severe accident (Liikenneturva, 2019).

Driving can be seen as being comprised of two psychologically distinct components, i.e., driver performance and behaviour, which both influence a driver’s likelihood of having a crash (Elander et al., 1993; Evans, 2006), and to information processing and motor skills, which should improve with practice and training (i.e., with driving experience). Shortcomings in driving performance result as errors and mistakes while driving. Driver behaviour refers to driving style (i.e., the way the driver chooses to drive), and includes such behaviours as speed choice, following traffic rules and readiness to drive while intoxicated or tired. A risky driving style is, thus, characterised by aberrant behaviours, which are strongly related to the probability of an accident. One of the most widely used self-report instrument for measuring aberrant driving behaviour is the Driver Behaviour Questionnaire (DBQ; Reason et al., 1990). In the DBQ, drivers are asked to indicate how often they have committed various aberrant behaviours while driving during the past year. These aberrant behaviours can be classified as errors, lapses, aggressive violations or ordinary violations. Errors can be defined as unwanted results of involuntary actions, whereas violations are based on conscious deviations from a rule or safe practice. Errors were further divided into slips, lapses and mistakes. Slips (attention deficits) and lapses (memory failures) are the results of cognitive processing problems. Later, violations were further divided into ordinary violations and aggressive violations (Lawton et al., 1997). In aggressive violations, the primary motivation is aggression, while ordinary violations are deliberate violations of traffic rules, or deviations from safe practices without aggressive intent. The DBQ factor structure has been studied extensively, indicating that the DBQ is comprised of two (errors and violations), three (errors, violations and lapses) or four factors (errors, ordinary violations, aggressive violations and lapses). The benefit of the four-factor structure used in the present study is that it separates aggressive violations from ordinary violations and gives a more accurate view of the motives behind driving violations. The DBQ has been used in many studies conducted in several countries, including Finland (Lajunen et al., 2004; Özkan et al., 2006, 2006a; Warner et al., 2011; Mattsson et al., 2015).

The main reasons for the higher accident rate among young drivers are inexperience associated with inadequate driving skills, and immaturity characterised by risk-taking (Shinar, 2017). Driving skills can be organised hierarchically into four levels: ‘goals for life and skills for living’, ‘goals and context of driving’, ‘mastery of traffic situations’ and ‘vehicle manoeuvring’ (Berg, 2006). The lowest levels of driving skills (i.e., vehicle manoeuvring) are acquired quickly, while it takes much more time and practice in traffic for novice drivers to develop the higher-order perceptual and information processing skills necessary to drive safely (Deery, 1999). Mueller and Trick (2012) compared experienced and inexperienced drivers in foggy conditions and found that experienced drivers adjusted their speed in foggy conditions more than inexperienced drivers (Mueller and Trick, 2012). Underwood et al. (2002) showed novice and experienced drivers’ video recordings taken from a car travelling along a variety of roads and recorded their eye movements to identify eye scanning patterns, as they followed instructions to indicate hazardous events (Underwood et al., 2002). The experienced drivers showed more extensive scanning patterns on the demanding sections of the dual-carriage way (Underwood et al., 2002). Similar findings demonstrating more developed information processing skills among experienced drivers have also been reported in several other studies (Borowsky et al., 2008, 2010; Lehtonen et al., 2014).

Driving is usually characterised by two motivation factors: mobility and safety. Drivers aim to arrive at their destination as smoothly and safely as possible. In this way, drivers have to find an optimal balance between mobility and safety. In addition to these two ‘rational’ motives of driving, drivers may also display so-called ‘extra motives’ (Summala, 1988; Salminen and Lähdeniemi, 2002), which are motives that are not directly related to arriving at their destination as quickly and safely as possible. Due to immaturity, young (mainly male) drivers have many non-driving related motives that influence their driving. Earlier studies have demonstrated that sensation, thrill and adventure-seeking increase risky driving, such as speeding (Cestac et al., 2011; Delhomme et al., 2012; Hatfield et al., 2014), driving while intoxicated (Dunlop and Romer, 2010; Hatfield et al., 2014; Luk et al., 2017; Navas et al., 2019) and reckless driving in general (Miller and Taubman-Ben-Ari, 2010; Hatfield et al., 2014; Eherenfreund-Hager and Taubman-Ben-Ari, 2016). These studies also show a clear difference between young men and women, which partly explains why young men are greatly overrepresented in crash statistics.

The main problem among young drivers, especially young male drivers, is the combination of inexperience and immaturity: the lack of skills interacts with reckless driving due to immaturity. Hence, it can be supposed that rather than vehicle handling skills, as such, the imbalance between safety skills and perceptual-motor skills leads to a higher crash risk. Sümer et al. (2006) hypothesised that the combination of self-reported high ratings of driving skills and low ratings of safety skills creates a higher risk for road crash involvement. Self-evaluated perceptual-motor and safety skills were measured using the Driver Skill Inventory (DSI; Lajunen and Summala, 1995). The DSI asks drivers to indicate the ‘strong and weak components’ in their driving, in terms of skills which represent either perceptual-motor skills (e.g., ‘fast reactions’ and ‘controlling the vehicle’) or safety skills (e.g., ‘driving carefully’ and ‘staying calm in irritating situations’). Sümer et al.’s (2006) results revealed that driving skills moderated the effects of safety skills on six out of the eight outcome variables, including the number of accidents, tickets, overtaking tendencies, speed on motorways and aggressive driving style.

The first aim of the present study was to investigate how age and driving experience (mileage) influence young (18–25 year olds) drivers’ self-assessed perceptual-motor and safety skills (measured by the DSI), as well as self-reported aberrant driver behaviour (measured by the DBQ), i.e., errors, ordinary violations, aggressive violations and lapses. The second aim was to study the relationships between self-assessed perceptual-motor skills, safety skills and aberrant driver behaviour, as a function of age and driving experience (mileage), in a cross-sectional sample of newly licenced young drivers.

## Materials and Methods

### Participants

The sample was collected as a stratified random sample from the driving licence register. A survey questionnaire was sent to 3,000 young car drivers. Participants were assured of the confidentiality of responses and were enrolled in a lottery for one of two €250 prizes, as incentives to participate.

The final dataset consisted of 1,058 completed questionnaires. The mean age of car drivers was 20.6 years (range: 18–29 years, standard deviation was 1.85 years) and 62.2% were female. Since there were only six respondents older than 25 years, all respondents aged 25 or older were included in one category labelled ‘age ≥25’. The mean lifetime mileage was 33,773 km (SD 67,767 km) and 8.4% reported that they had driven at least once for work.

### Materials

#### The Driver Behaviour Questionnaire

The Finnish translation of the 28-item DBQ (Lawton et al., 1997; Lajunen et al., 2004) was used in the current study. The item related to driving under the influence of alcohol was removed, as recommended by Lajunen et al. (2004), due to the very low variance found in that item and the fact it does not load on any of the factors (e.g., Sullman et al., 2002). Thus, the final 27 item version of the DBQ included ‘errors’ (8 items), ‘lapses’ (8 items), ‘ordinary violations’ (8 items) and ‘aggressive violations’ (3 items). The self-reported behaviours in the previous year were recorded on a six-point Likert scale (1 = never and 6 = nearly all the time).

#### The Driver Skill Inventory

The DSI is a 20 item self-reported measure of perceptual-motor (11 items; e.g. fluent driving) and safety skills (9 items; e.g. conforming to the speed limits; Lajunen and Summala, 1995). The DSI was initially developed in Finnish and has been used in many studies in Finland (Lajunen et al., 1998a,b; Özkan and Lajunen, 2006; Özkan et al., 2006b; Öz et al., 2013; Warner et al., 2013). In the DSI, drivers are asked to rate how weak or strong they feel they were in each given skill, using a 5- point Likert scale (1 = very weak and 5 = very strong).

#### Demographic Measures

Respondents answered questions about their age, sex, the number of years they had held a full driving licence and their lifetime mileage.

### Statistical Analyses

Sex differences on the DBQ and DSI subscales were analysed using *t*-tests. A Multivariate analysis of variance (MANOVA) was used to investigate the effects that age group, sex and lifetime mileage had on the DSI subscales (i.e., safety skill and perceptual-motor skill scores), as well as the DBQ subscales (i.e., ordinary violations, aggressive violations, errors and lapses). Univariate ANOVAs were used to test for interaction effects. All analyses were performed using SPSS (Statistical Package for Social Sciences) version 25.

## Results

### The Overall Scale Score Differences Between Men and Women

The means and standard deviations for the DBQ and DSI scales can be found in Table 1. In general, women scored lower than men on aggressive and ordinary violations, as well as on self-reported perceptual-motor skills. However, women reported more lapses and higher safety skills than men, but no difference was found for errors.

**Table 1:** Scale reliability coefficients (both sexes included), means (*M*), and standard deviations (SD) for men and women as well as independent samples *t*-test results comparing men and women.

		Woman		Man		
	*α*					*t*-test
		*M*	SD	*M*	SD	
Aggressive violations	0.75	1.39	0.60	1.63	0.80	−5.55^*^
Ordinary violations	0.77	1.93	0.50	2.20	0.63	−7.75^*^
Lapses	0.62	2.01	0.46	1.85	0.45	5.20^*^
Errors	0.69	1.47	0.35	1.49	0.39	−0.88
Perceptual-motor skills	0.88	3.12	0.56	3.73	0.62	−16.55^*^
Safety skills	0.82	3.56	0.57	3.31	0.64	6.70^*^

* *p < 0.001*.

*df’s range in t-tests: 958–1049*. *t-test categories: man = 1; woman = 2*.

### The Effect of Age and Driving Experience (Mileage) on Perceptual-Motor and Safety Skills

The MANOVA showed that the respondents were placed into eight age groups (18; 19; 20; 21; 22; 23; 24; and ≥25 years) and three groups according to lifetime mileage (≤5,000 km; >5,000—24,900 km; and ≥25,000 km). Lifetime mileage was used as a categorical variable, not as a covariate, in order to calculate the interaction effects with sex and age. Hence, the following main effects, two-way interactions and three-way interactions were tested as: Age; Sex; Mileage; Age × Sex; Age × Mileage; Sex × Mileage; and Age × Sex × Mileage.

For clarity, only statistically significant MANOVA (GLM in SPSS) and univariate ANOVA results are listed in Table 2. The results showed significant multivariate effects for age group, sex and lifetime mileage. In addition, interaction effects were found for age × sex, age × lifetime mileage, but not for sex × lifetime mileage. A three-way interaction (age × sex × driving experience) effect was also found (Table 2).

**Table 2:** MANOVA and ANOVA results for the DSI and the DBQ.

Variable	Wilks’ lambda	*F*	df	*η* ^2^
MANOVA: Perceptual-motor skills and safety skills (DSI)				
Age	0.95	3.34^***^	14,1810	0.03
Sex	0.94	30.71^***^	2,905	0.06
Mileage	0.89	26.40^***^	4,1810	0.06
Age × Sex	0.97	1.77^*^	14,1810	0.01
Age × Mileage	0.95	1.63^*^	28,1810	0.03
Age × Sex × Mileage	0.96	1.60^*^	26,1810	0.02
ANOVA: safety orientation				
Sex	–	40.10^***^	1,906	0.04
Age	–	5.13^***^	7,906	0.04
Mileage	–	46.49^***^	2,906	0.09
MANOVA: Aberrant driving behaviour (DBQ)				
Sex	0.97	6.55^***^	4,904	0.03
Mileage	0.96	4.75^***^	8,1808	0.02
Age × Mileage	0.92	1.44^*^	56,3519	0.02
ANOVA: Ordinary violations (DBQ)				
Sex	–	9.24^**^	1,907	0.01
Mileage	–	12.35^***^	2,907	0.03
Age × Mileage	–	1.95^*^	14,907	0.03
ANOVA: Aggressive violations (DBQ)				
Mileage	–	11.40^***^	2,907	0.03
ANOVA: Lapses (DBQ)				
Sex	–	5.55^*^	1,907	0.01
Age × Mileage	–	1.80^*^	14,907	0.03

*Effects tested were Age; Sex; Mileage; Age × Sex; Age × Mileage; Sex × Mileage; and Age × Sex × Mileage*.

* *p < 0.05*;

** *p < 0.01*;

*** *p < 0.001*.

**Figure 1:**
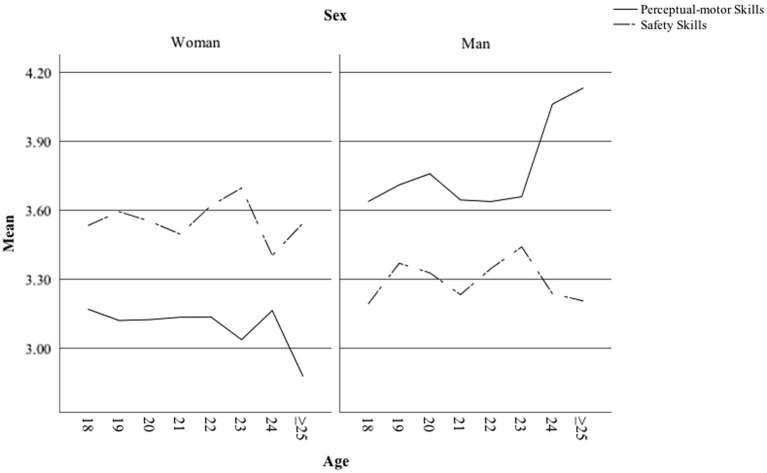
Perceptual-motor and safety skills as a function of age among men and women.

The relationships between age, perceptual-motor and safety skills for both sexes are shown in Figure 1. Figure 1 shows age differences in perceptual-motor and safety skills among men and women. While female drivers assessed their safety skills more highly than their perceptual-motor skills, the opposite was true for males. Among men, age was positively related to self-evaluated perceptual-motor skills, while among women self-reported perceptual-motor skills were not evaluated as higher among older respondents.

**Figure 2:**
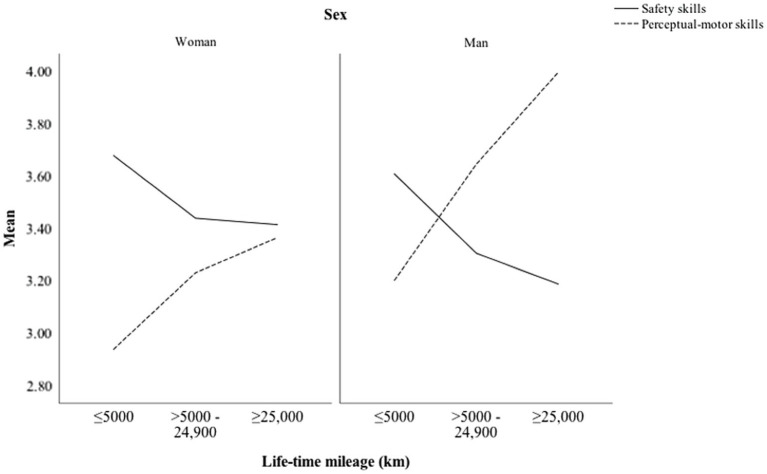
Perceptual-motor and safety skills as a function of lifetime mileage among men and women.

Figure 2 shows the self-assessed perceptual-motor and safety skills as a function of driving experience (lifetime mileage) among men and women. In both sexes, the perceptual-motors skills increased, and safety skills decreased with higher levels of experience. The effect was much more pronounced among men than women and also in the first 5,000 km.

**Figure 3:**
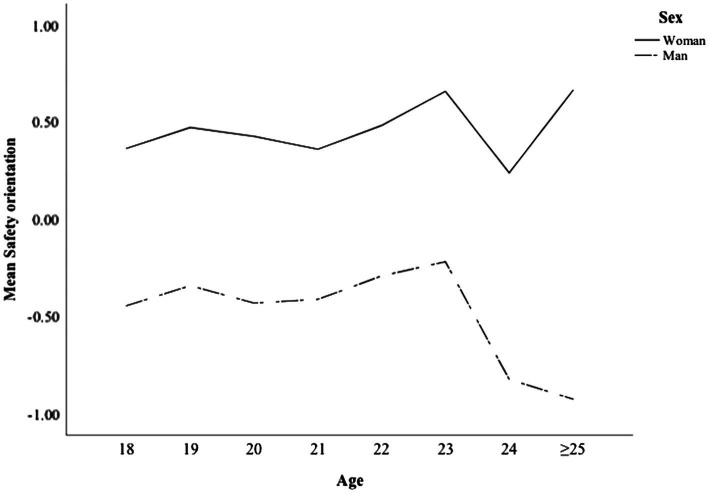
Safety orientation as a function of age among men and women.

In the DSI, drivers are asked to assess their safety and perceptual-motors skills in relation to each other (‘Which are the strong and weak components of your driving?’). As pointed out by Lajunen and Summala (1995), it is more important to assess the difference between perceptual-motor skills and safety skills than the individual score. The ‘safety orientation score’ (perceptual-motor skills minus safety skills) was calculated to measure drivers’ emphasis on safety instead of vehicle handling skills. Figure 3 shows the safety orientation among men and women, as a function of age group.

The Univariate ANOVA analysis showed statistically significant main effects for sex, age group and driving experience (mileage; Table 2). None of the interactions (sex × age, sex × mileage, age x mileage and age × sex × mileage) were significant. The results show clearly that women scored higher in safety orientation than men. Interestingly, among men, safety orientation was lower in those aged over 23 years of age.

**Figure 4:**
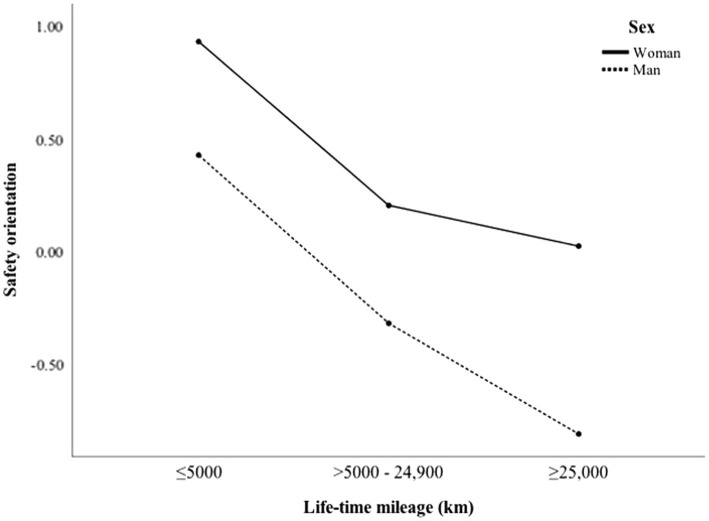
Safety orientation as a function of lifetime mileage among men and women.

Figure 4 presents the level of safety orientation according to licence tenure. Figures 3 and 4, also show that women scored higher in safety orientation than men in all driving experience groups. More interestingly, among both men and women, experienced drivers scored much lower in safety orientation than inexperienced drivers. While those with more experience had higher confidence in their perceptual-motor (i.e., vehicle handling) skills, more experienced drivers reported lower safety skills.

### The Effect of Age, Sex, and Driving Experience (Mileage) on Aberrant Driving Behaviour

**Figure 5:**
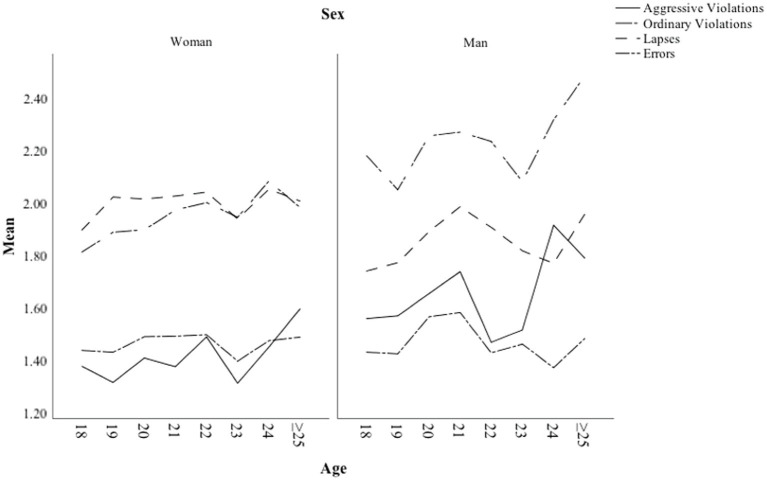
Aggressive violations, Ordinary violations, Lapses and Errors as a function of age among men and women.

**Figure 6:**
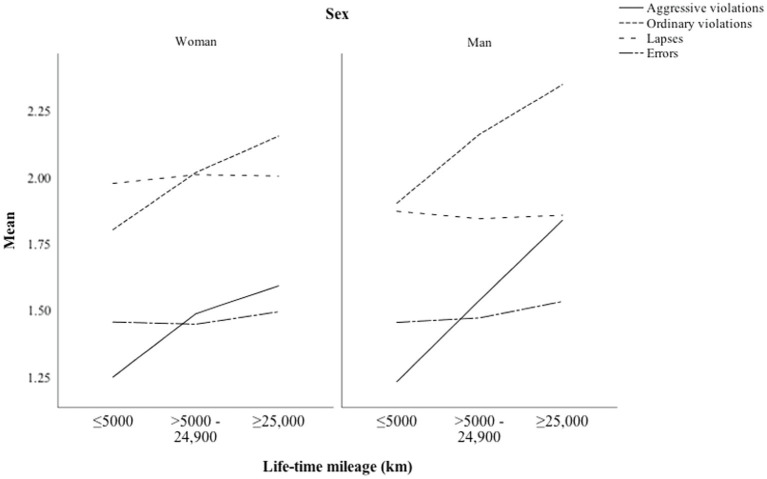
Aggressive violations, Ordinary violations, Lapses and Errors as a function of lifetime mileage among men and women.

A multivariate analysis of variance (MANOVA) was used to investigate the effect of age group, sex and lifetime mileage on aberrant driving behaviour (i.e., aggressive violations, ordinary violations, lapses and errors). These relationships are pictured in Figures 5, 6. The MANOVA (GLM in SPSS) results showed significant multivariate effects for sex and mileage, whereas no main effect was found for age. An interaction effect for age and mileage was found (Table 2), whereas no interaction effects were found for sex and age, sex × mileage or age × sex × mileage.

The three-way (sex × age × mileage) univariate ANOVA showed sex differences in ordinary violations and lapses (Table 2), but not in aggressive violations or errors. The analyses did not show the main effects of age in any of the DBQ variables. The main effects of mileage were found on aggressive violations and ordinary violations (Table 2) but not for errors or lapses. In terms of interactions, the only statistically significant interactions found were the age × mileage interaction on ordinary violations and lapses (Table 2).

### The Relationships Between DSI Perceptual-Motor Skills and Safety Skills

According to Lajunen and Summala (1995), a driver’s view of his or her driving skills is composed of perceptual-motor skills and safety skills. Pearson product–moment correlation coefficients between background variables (age, sex and mileage), perceptual-motor skills and safety orientation score can be seen in Table 3. Table 3 shows that being male correlated positively with perceptual-motor skills and negatively with safety skills, while age had no correlations with the DSI variables. Perceptual-motor skills correlated negatively with safety skills, DBQ lapses and errors, and positively with DBQ aggressive and ordinary violations. Safety skills correlated negatively with all DBQ aberrant driving variables. Similar correlations can be seen between safety orientation, sex, mileage and DBQ subscale scores. It should be noted that safety orientation had moderate correlations with both DBQ violation subscales, while the correlations with mistakes (errors and lapses) were minimal. Safety orientation seems to be reflected mainly in deliberate aberrant behaviours, (i.e., ordinary and aggressive violations).

**Table 3:** Correlations between background variables, self-evaluated perceptual-motor skills, safety skills and safety orientation.

	1	2	3	4	5	6	7	8	9
1. Age	1.00								
2. Sex	−0.03	1.00							
3. Lifetime Mileage (km)	0.23^***^	0.18^***^	1.00						
4. Perceptual-motor Skills	0.00	0.46^***^	0.27^***^	1.00					
5. Safety Skills	0.01	−0.20^***^	−0.16^***^	−0.26^***^	1.00				
6. Safety orientation	0.00	−0.42^***^	−0.27^***^	−0.81^***^	0.78^***^	1.00			
7. Aggressive Violations	0.06^*^	0.17^***^	0.22^***^	0.34^***^	−0.51^***^	−0.53^***^	1.00		
8. Ordinary Violations	0.09^**^	0.23^***^	0.16^***^	0.30^***^	−0.60^***^	−0.56^***^	0.53^***^	1.00	
9. Lapses	0.05	−0.16^***^	−0.02	−0.23^***^	−0.12^***^	0.07^*^	0.13^***^	0.33^***^	1.00
10. Errors	0.01	0.03	0.02	−0.13^***^	−0.26^***^	−0.07^*^	0.24^***^	0.38^***^	0.52^***^

* *p < 0.05*;

** *p < 0.01*;

*** *p < 0.001*.

**Figure 7:**
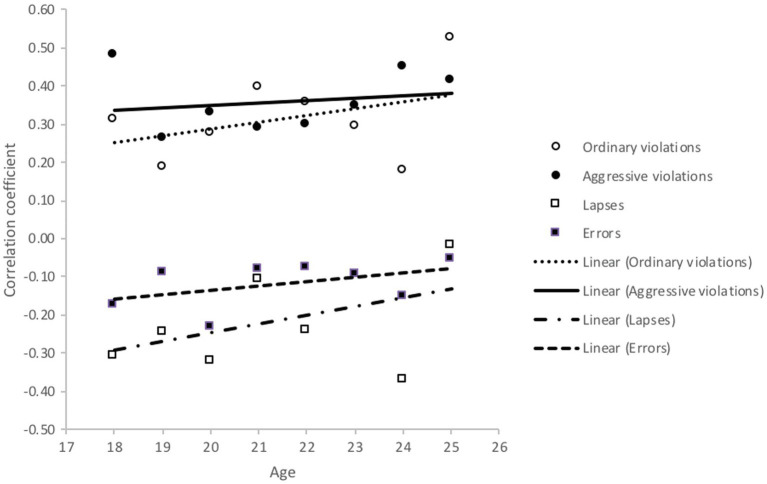
Correlations between aberrant driver behaviours (DBQ) and perceptual-motor skills (DSI) by age.

**Figure 8:**
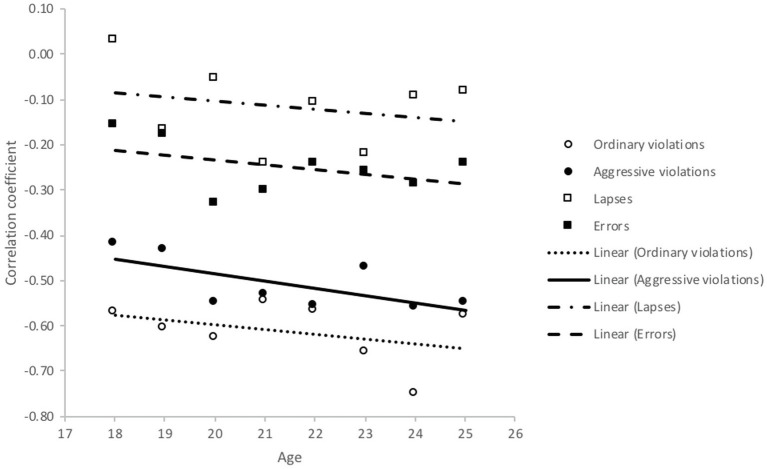
Correlations between aberrant driver behaviours (DBQ) and safety skills (DSI) by age.

The correlations between self-evaluated skills (perceptual-motor and safety skills) and aberrant driving behaviour were calculated separately for each age group. These correlations are presented in Figures 7, 8. In general, it can be said that despite some obvious random fluctuations among age groups, the correlations between self-evaluated driving skills (perceptual-motor and safety skills) were constant across the ages and followed the pattern described in Table 3. As Figure 7 shows, the correlations between perception-motor skills and violations (especially ordinary violations) got stronger with age. On the other hand, the correlation between perception-motor skills and mistakes (especially lapses) got weaker. This finding might indicate that the role of perceptual-motor skills gets stronger in the first 5 years of driving, while the relationship between perceptual-motor skills and indeliberate errors becomes less pronounced.

Figure 8 shows almost the opposite developments in the relationships between skills and aberrant driving behaviours. Safety skills had stronger negative correlations with ordinary and aggressive violations among older (but still young) drivers. The same phenomenon was also evident in the correlations that safety skills had with errors and lapses.

**Figure 9:**
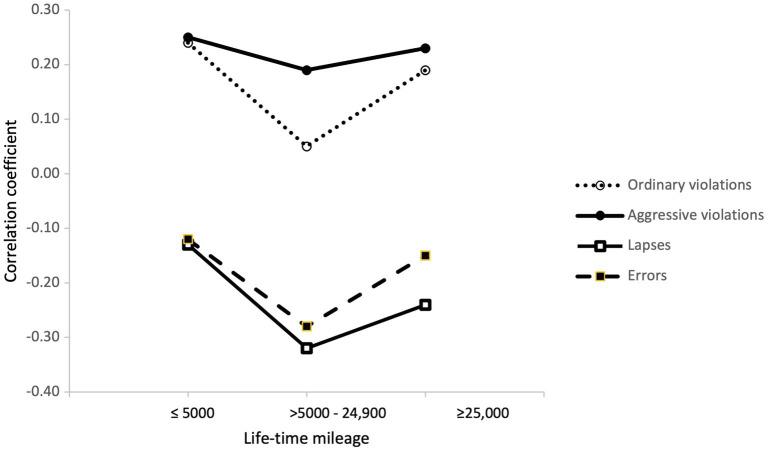
Correlations between aberrant driver behaviours (DBQ) and perceptual-motor skills (DSI) in the mileage groups.

**Figure 10:**
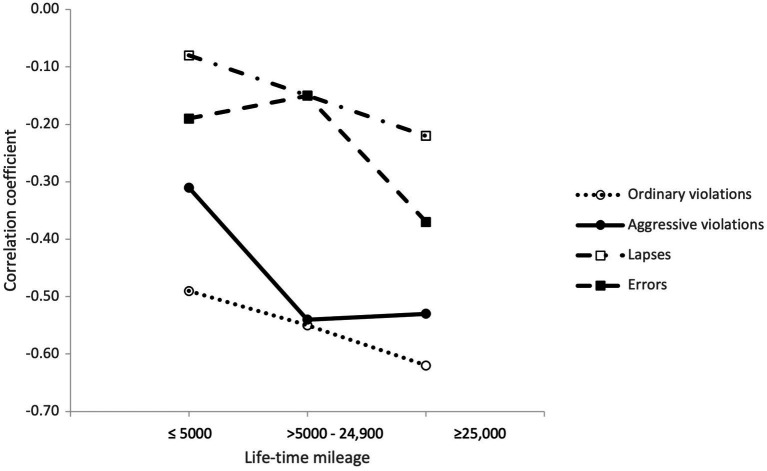
Correlations between aberrant driver behaviours (DBQ) and safety skills (DSI) in the mileage groups.

Figures 9, 10 show the correlations the different types of aberrant driver behaviours had with the DSI subscales of perceptual-motor skills and safety skills, respectively. Figure 9 shows that the mileage group with a lifetime mileage between 5,000 and 24,900 km was particularly different from inexperienced (≤5,000 km) and more experienced (≥25,000 km) drivers. The correlation between perceptual-motor skills and ordinary violations was clearly lower in this mileage group, than was the case among the less experienced and more experienced drivers. On the other hand, the correlation (negative) between perceptual-motor skills and errors was stronger in this group than among the less and more experienced drivers.

The correlations between aberrant driver behaviours and safety skills followed a somewhat different pattern, in terms of driving experience (Figure 10). The negative correlations between safety skills and aberrant driver behaviours, especially with both types of violations, seemed to get stronger with increasing driving experience. This indicates that safety skills (i.e., emphasis on safe behaviours) might be even more crucial for avoiding aberrant driving behaviour among experienced than inexperienced drivers.

## Discussion

The main aim of the present study was to examine self-evaluated driver safety and perceptual-motor skills in different groups of young drivers (18–25 year olds), along with the relationships between self-evaluated safety skills and driving behaviour. In general, women reported more lapses than men, but less aggressive and ordinary violations. These sex differences are similar to those reported elsewhere in research using the DBQ (e.g., Gras et al., 2006; Sullman et al., 2019). However, what may appear surprising is the absence of a multivariate main effect for age on aberrant driving behaviour, but since our research only included a very narrow age range, the age differences would be expected to be low. In contrast, a multivariate main effect for mileage on aberrant driving behaviour was found. Previous research using the DBQ has also reported experience and age-specific changes in errors and violations for young drivers (e.g., De Winter and Dodou, 2010). Specifically, self-reported violations have been found to increase with driving experience (despite the opposite tendency in older drivers), while error scores rapidly decrease with driving experience and then remain relatively stable across most age and driving experience groups (De Winter and Dodou, 2010). The findings of the present study also agree with previous research on crashes, which have found experience to be a bigger contributor to crash risk than age among young drivers (e.g., Maycock et al., 1991; Forsyth et al., 1995; Vlakveld, 2004). In particular, research has found that crash risk declined much more rapidly as a result of experience than as a result of increased age (e.g., Vlakveld, 2004).

The present study also found age and sex differences for the DSI subscales, with women self-reporting lower perceptual-motor skills than men, but higher safety skills. Furthermore, female drivers assessed their safety skills to be higher than their perceptual-motor skills, while the opposite was true for males. Age was also positively related to self-evaluated perceptual-motor skills among males, but did not differ by age among females. In addition, the present study also showed that women had a higher safety orientation score than males and that the safety orientation scores for males reduced sharply after the age of 23. In both men and women, perceptual-motors skills were higher in those with more experience, while safety orientation decreased with higher lifetime mileage, for both men and women. These findings demonstrate why these young drivers are particularly at risk of crashing. Particularly problematic is the tendency for young male drivers to highly rate their own skills for controlling and manoeuvring the vehicle, in comparison with young females, but to also report lower ‘higher level’ safety skills than females. It is likely that this is one of the reasons young males have much higher crash rates than young females.

In terms of the relationship between the DSI and DBQ variables, perceptual-motor skills were positively correlated with violations and aggressive violations. In contrast, lapses and errors were negatively correlated with perceptual-motor skills. Therefore, drivers who have lower perceptual-motor skills are more likely to also report more errors and lapses, while those who report that they are more skilful drivers are more likely to engage in aberrant driving behaviours (i.e., ordinary violation and aggressive violations). Furthermore, safety skills were negatively correlated with all DBQ variables, indicating that those reporting higher safety skills were less likely to also report engaging in errors, lapses, violations and aggressive violations. These findings are similar to those reported using a large sample of drivers from the Danish population (18–84 years old), except that they found a positive relationship between violations and perceptual-motor skills (Martinussen et al., 2014).

The present research suggests that drivers’ assessment of their own driving skills is reflected in their driving style, or more specifically their engagement in aberrant driving behaviours. Those drivers who report low levels of driving safety skills, also report more frequent engagement in aberrant driving behaviours. The observed mismatch between the young drivers’ perceptions of their own skills and abilities has important implications for their risk perceptions and risk-taking behaviour, as well as for the development of interventions. Although the present study did not measure the relationship these variables had with crash involvement, it is clear that young drivers who perceive themselves as having high levels of perceptual-motor skills, and also have low safety skills, are most at risk of being crash involved. The results of the present study also suggest that drivers should undergo training to allow them a more accurate understanding of their actual driving skills and to reduce any overconfidence or false sense of security. The use of training to reduce overconfidence bias has been raised previously (Özkan et al., 2006b) and is clearly justified by the present findings, particularly regarding young males.

This study shows that overconfidence in one’s perceptual-motor skills, together with a low regard for safety skills, were more apparent in those with greater driving experience and that overconfidence was also correlated with both aggressive and ordinary violations. This effect might be even stronger in reality, than in a study based on self-assessments and self-reports. In terms of novice driver education and policy, this finding means that special programmes which focus on safety attitudes, realistic self-assessments and safe driving practices should be applied in novice driver training. Focusing on vehicle handling and manoeuvring skills leads only to the development of overconfidence. According to a recent study by Rodwell et al. (2021), the most promising Graduated Driver Licencing systems are those in which parents play an important role in teaching their children how to drive. These programmes often follow a holistic framework called the Goals for Driver Education (GDE; Berg, 2006), which groups influence on young drivers’ behaviour into four interconnected hierarchical levels: vehicle manoeuvring (Level 1), mastery of traffic situations (Level 2), goals and contexts of driving (Level 3) and goals for life and skills for living (Level 4; Berg, 2006). According to Rodwell et al. (2021), the most effective combination could be education programmes in which professional driving instructors teach levels 1 and 2, while parents teach their child levels 3 and 4, in which the learner driver should learn safe attitudes to driving. An essential part of the graduated driver licencing programmes used in North America, Australia and New Zealand is a ‘learner’ stage, where young drivers are supervised by an experienced driver, usually a parent. This kind of parent supervised driving is an excellent opportunity for teaching the learner driver level 3 and 4 ‘skills’ including safe attitudes and a realistic assessment of one’s own driving, i.e., safety orientation, which the DSI measures. While level 1 and 2 skills (perceptual-motor skills) are learned fast, the formation of safe attitudes needs much more time and, therefore, are best learned while driving with a responsibly experienced driver, such as a parent. The results of the present study, based on more than a thousand young drivers, give strong support to the GDE framework, and also graduated driver licencing programmes, which are based on the GDE approach.

While the present study is only based on comparisons between age and driving experience groups, and not using a longitudinal design, the results may indicate that inexperienced and young drivers could benefit from a mandatory follow-up training programme, in which the safety skills and safe driving style could be reinforced. Since the (over) confidence in vehicle handling skills is much higher in those with more driving experience, while the opposite is true regarding concern for safety, it may be beneficial to require drivers to participate in a mandatory follow-up training programme 2 years after licencing. This short programme could include a self-assessment of driving, together with objective measures of safety, which could be used to show the drivers their real skill level. Combined with well-designed simulations, the training programme—even a training day—could serve as a healthy reminder about the risks of overconfidence in traffic.

The present study was based on a stratified random sample of young drivers from the licence register, meaning that the sample should be representative of young Finnish drivers. However, this study also has some limitations, which should be taken into account. Firstly, the representativeness of surveys may be greatly limited by the low response rate, which are often seen in postal surveys. As Lajunen and Özkan (2011) pointed out, the most reckless drivers who frequently commit traffic violations (as asked in the DBQ) are not necessary the most keen respondents to postal surveys. Therefore, the present study might not have captured those young drivers who commit the highest number of traffic violations. Secondly, as the data were all self-reported this raises the prospect that social desirability bias may have influenced the findings. While it is clear that social desirability bias will have had some impact upon the results, as the participants were assured that the answers they provided were completely confidential, the impact is likely to be low. Furthermore, under these conditions research evidence for the DBQ has found the impact of social desirability bias to be mostly insignificant (Lajunen and Summala, 1995; Sullman and Taylor, 2010). However, as both the dependent and independent variables were measured using self-report, common method variance may have inflated some of the relationships reported here. Thirdly, the study used a cross-sectional design, in which different age and driving experience groups were compared. While we can assume that comparisons of group means indicate the effect of age and driving experience on driving style and drivers’ self-evaluated driving skills, the cross-sectional between-subjects design does not allow causal explanations. In future, follow-up studies using within-subjects design are needed. The best approach would be to follow the same drivers from the first kilometres onwards, throughout their driver career. While this kind of large long-lasting follow-up study is difficult to conduct and requires considerable resources, they are the only means to accurately describe how driving style and skills develop as a function of driving experience.

## Data Availability Statement

The raw data supporting the conclusions of this article will be made available by the authors, without undue reservation.

## Ethics Statement

Ethical review and approval was not required for the study on human participants in accordance with the local legislation and institutional requirements. The patients/participants provided their written informed consent to participate in this study.

## Author Contributions

TL, MS, and EG contributed to the conception and design of the study, performed the statistical analysis, and wrote sections of the manuscript. TL organized the database. TL and MS wrote the first draft of the manuscript. All authors contributed to the article and approved the submitted version.

## Conflict of Interest

The authors declare that the research was conducted in the absence of any commercial or financial relationships that could be construed as a potential conflict of interest.

## Publisher’s Note

All claims expressed in this article are solely those of the authors and do not necessarily represent those of their affiliated organizations, or those of the publisher, the editors and the reviewers. Any product that may be evaluated in this article, or claim that may be made by its manufacturer, is not guaranteed or endorsed by the publisher.
